# Protocol paper for an implementation science approach to promoting colorectal cancer screening in Federally Qualified Health Center clinics: A stepped-wedge, multilevel intervention trial

**DOI:** 10.21203/rs.3.rs-4558718/v1

**Published:** 2024-07-18

**Authors:** Samantha M. Montoya, Pelumi Olaore, Janna Bastardo-Acosta, Rania Abdulla, Michael J. Schell, Allan Hidalgo, Barbara Turner, Monica Rider, Nirmala Kishun-Jit, Ashlon Joshua, Jennifer Pollard, Mark Friedman, Shannon M. Christy, Cathy D. Meade, Clement K. Gwede

**Affiliations:** Moffitt Cancer Center; University of South Florida; University of South Florida; Moffitt Cancer Center; Moffitt Cancer Center; Central Florida Health Care; Central Florida Health Care; MCR Health; MCR Health; MCR Health; MCR Health; Moffitt Cancer Center; Moffitt Cancer Center; Moffitt Cancer Center; Moffitt Cancer Center

**Keywords:** colorectal cancer screening, Federally Qualified Health Centers, implementation science, randomized stepped-wedge intervention trial

## Abstract

**Background:**

Colorectal cancer is the third leading cause of cancer-related deaths in the United States. Despite the Healthy People 2030 goal of 70.5%, colorectal cancer (CRC) screening rates in Federally Qualified Health Centers (FQHCs) are suboptimal at about 40%. The Colorectal Cancer Awareness, Research, Education and Screening-Rural Expansion, Access, and Capacity for Health (**CARES-REACH**) study seeks to address this disparity and accelerate the adoption and utilization of effective, evidence-based CRC screening practices. This paper describes the CARES-REACH study design and implementation methods.

**Methods:**

Informed by a community-based participatory research (CBPR) framework and enriched by implementation science approaches, CARES-REACH features a stepped wedge design with extension for maintenance to support an implementation strategy focused on multiple levels: organizational, provider, and patient levels that entail processes to boost initial and repeat screening among average risk and age-eligible adults. This multilevel study entails the implementation of a core set of evidence-based interventions (EBIs) that include low literacy patient education (English, Spanish, and Haitian Creole language); provider education, system-wide electronic medical record (EMR) tools including provider prompts and patient reminders, FIT (fecal immunochemical test) kit distribution, plus an organization-wide cancer control champion who motivates providers, coaches and navigates patients, and monitors system-wide CRC screening activities.

**Trial registration:**

NCT04464668.

## INTRODUCTION

In 2023, approximately 153,020 new cases of colorectal cancer (CRC) were diagnosed in the United States (US), making it the third most common cancer diagnosed in both men and women (1). Regular screening of this detectable cancer offers an excellent opportunity to enhance the health outcomes of patients and community members, especially individuals who are underinsured or uninsured and being cared for in medical homes such as Federally Qualified Health Centers (FQHCs). The CRC screening rates in FQHCs are below both the national Health People targets (Healthy People 2020 target: 70.5% or Healthy People 2030: 74.4%). This lower performance may be attributed to a wide range of factors, such as lower rates of participation in screening due to unequal access or lack of health insurance, less access to screening/early detection and early treatment, and transportation obstacles, among others (2–6). Thus, the implementation of real-world interventions strategically placed in community-based clinics is needed to achieve a national goal of 80% in every community (7).

Both the US Preventive Services Task Force (USPSTF) and the American Cancer Society have recently updated recommendations for asymptomatic adults at average CRC risk from age 50–75 to age 45–75. Individuals at average CRC risk can be screened with multiple modalities, including annual stool-based tests (such as fecal immunochemical test [FIT]), or stool DNA tests [e.g., Cologuard] every three years, colonoscopy every ten years, CT colonography every five years, and/or flexible sigmoidoscopy every five years (8, 9). The use of FIT as a primary screening modality reflects a critical pragmatic approach, which is highly endorsed in low-resource environments such as FQHCs due to the limited availability of colonoscopy (10, 11). FIT is also often more accessible to geographically dispersed communities and at a lower cost (compared with colonoscopy) (12). In Florida, FQHC screening rates for patients aged 50–75 were 47% in 2019 and declined to 40% percent in 2020. Similarly, screening rates were 45% nationally in 2019 and dropped to 40% in 2020 (13, 14).

Recent studies, including our team’s preliminary investigations in community clinics (15–17), have demonstrated that CRC screening using a single-application FIT is well received by both patients and providers and can lead to initial screening rates of 80% (15–26). Our prior work has demonstrated high initial FIT uptake and endorsed the use of FIT paired with EBIs, such as videos, brochures, etc. (15–17, 27). Further, including coaches/navigators often increases patient engagement, mitigates obstacles, and serves as a way to bring resources for improving health outcomes for chronic diseases (54, 55). Attention to culture, literacy, and language (including English, Spanish, and Haitian Creole) to support CRC educational messaging is also a prerequisite for effective patient education. (56, 57, 58). The evidence-based interventions (EBIs) in CARES-REACH are based on characteristics and components that have been shown to be feasible and effective, such as using electronic health records (EHR) to identify patients needing screening and providing culturally, linguistically, and literacy-level-appropriate information in a clear manner (28). Evidence-based interventions, such as providing accessible FIT kits, have also been supported by several studies as an approach to removing access obstacles (10, 11, 29). Other effective educational EBIs include patient-directed videos, pamphlets, reminder letters/messages, and provider/nursing reminders and prompts (28).

FIT requires annual repeat testing to optimize clinical benefits (30–35), but repeat screening rates often decline sharply following initial screening (22%–60%) (19, 20, 36). This highlights the importance of implementing evidence-based intervention strategies to encourage both initial and repeat screening. However, multiple intersecting influences can impact the implementation of evidence-based practices in clinic settings. Strategies in prior studies were heavily led by the research team (15, 37–40); therefore, the next step is a more pragmatic trial with an implementation science-based approach. As such, a pressing gap remained to expand this work to broader geographically dispersed populations, including rural locations that may benefit from cancer screening. Therefore, our ‘real world’ implementation project, CARES-REACH, is intended to address this gap and assess intervention impact on annual FIT adherence over time.

The current paper describes the study protocol for the CARES-REACH intervention study, which is aimed to promote CRC screening among patients with medical homes at FQHCs, ages 45–75, and guided by implementation science approaches. The CARES-REACH study is a 5-year, multilevel intervention that leverages multiple evidence-based strategies and is fueled by community-based participatory research (CBPR) principles and community engagement approaches (15, 16, 41). In this paradigm, a Cancer Control-Implementation Advisory Board (CC-IAB) provides leadership and establishes decision-making procedures concerning the implementation of research activities by monitoring relevance, meaning, and overall practical utility. These perspectives are informed by our methodological research approach of **implementation science** whereby solutions for health disparities are gained through community and clinical member involvement, adding relevancy to the processes being implemented in ways that are salient, practical, scalable, and sustainable (42).

Selected constructs of the Reach, Effectiveness, Adoption, Implementation, and Maintenance (RE-AIM) framework (43) and Consolidated Framework for Implementation Research (CFIR) (44) further guide implementation evaluation of external and internal influences for this multilevel intervention to inform dissemination. RE-AIM constructs are used to characterize who is successfully reached by the intervention, the effectiveness of the intervention (uptake), adoption by the providers and system, implementation outcomes, and maintenance/sustainability.

The **scientific premise** of CARES-REACH is that an organization-wide multi-level CRC screening program can address persistent disparities in CRC mortality and screening rates, especially in FQHCs. The CARES-REACH study aims are as follows: (1) Implement an organization-wide multilevel CRC screening intervention plus a cancer control champion, (2) Explore whether practice settings and population characteristics impact annual CRC UDS screening rates, and (3) Conduct a comprehensive evaluation of the implementation process requirements, and intermediate patient screening outcomes. Study level components include Organization/Systems Level, Provider Level, and Patient level, with the Cancer Control Champion interacting at each intervention level. Various intervention strategies occur at each level and are depicted further in Table 1. The RE-AIM framework (43), and CFIR (44) guide implementation evaluation. **Findings** are expected to lead to a better understanding of the translation of research-tested intervention strategies in FQHCs and the processes that optimize (or hinder) intervention implementation under less controlled conditions.

## METHODS

### Study Design

Informed by implementation science and community-engaged approaches, CARES-REACH is a multilevel, multicomponent intervention featuring a stepped-wedge rollout and multilevel design (45, 46). The clinics (seven from each of the two Florida FQHC organizations) are randomized into two Waves (stratified by rural vs. urban location) and balanced by the number of patients 45–75 years [clinic volume] described in Table 2. Specifically, Wave 1 interventions were randomly selected to occur at four clinics in one FQHC organization. Then, precisely two of the four rural clinics, the smallest urban clinic and one of the two larger urban clinics, were randomly selected from that organization. An analogous approach was then used for the FQHC organization with four clinics in Wave 2 (three rural and four urban clinics). The CARES-REACH study supports implementing multiple strategies focused on the organizational, provider, and patient levels of influence to achieve the study aims described in Table 3.

### Setting

The setting for the CARES-REACH implementation is two FQHC organizations, which serve urban and rural regions in Central and Southwest Florida and provide care to diverse populations regardless of insurance status or ability to pay for services. The two FQHC organizations have each identified seven clinics, a mix of rural and urban-based clinics. Together, this allows us to assess whether the planned intervention has differential outcomes in two practice settings involving 14 clinics combined, with seven rural vs. seven urban clinics.

### Intervention Strategies

#### Organization/Systems Level:

We leverage existing EMR systems to support the patient-level intervention, laying the groundwork for future dissemination and implementation (18, 19, 47–52). The two clinic organizations use the same robust Athena Health Systems (Athenahealth^®^) EMR system, providing an exceptional opportunity to examine tracking features and capabilities as part of their organization’s response to National Committee on Quality Assurance (NCQA) and Patient-Centered Medical Homes requirements (53). These EMR systems allow for the collection of discrete data fields for CRC screening and related variables that can be directly downloaded (e.g., type of screening test, completion date, result). Essentially, three core components of the EMR systems can be leveraged to meet Aim 1: (1) the automated query feature to view screening status and results to identify and prioritize patients who are due for CRC screening; (2) documentation of discussion and ordering of CRC screening tests, and (3) automated calls, text messaging, and patient portals as ways to send patient reminders once patients receive an order for CRC screening (e.g., handed a FIT kit, or script/referral for colonoscopy).

### Provider Level

At the provider level, providers of various levels, including clinicians, nurses, and support staff, receive training offered by members of the research team in collaboration with the cancer control champion. Training topics included the importance of prioritizing CRC screening, current CRC guidelines, CRC screening options, use of the EMR to identify and track patients, delivery of compelling low-literacy screening messages, the importance of repeat screening, and EBIs (tools and resources). The cancer control champion also works directly with individual clinics and providers to support enhanced CRC screening intervention efforts.

### Patient Level

Providers deliver a core set of EBIs, including one-to-one patient education supported by low literacy/language-specific materials/media and recommending CRC screening (e.g., recommending FIT kit, Cologuard, or direct referral to colonoscopy as indicated). To best integrate this project into the workflow of the clinics, the core patient education intervention builds on existing and successful procedures for initial screening, repeat screening, and timely follow-up care, depending on screening modalities completed. When a FIT kit is provided, patients are instructed (in their preferred language) to collect the sample at home within 7–10 days and return (in person or by mail) to the clinic.

### Cancer Control Champion

A key component of CARES-REACH is the cancer control champion hired by each FQHC to work throughout the organization from a central location while leveraging the system-wide communication technology and the EMR. This centralized role expands the potential reach of CRC screening interventions, especially in settings such as FQHCs, where resources and efforts to improve cancer-related inequalities might be limited at individual clinic sites. The Cancer Control Champions have been hired and trained and bring backgrounds in healthcare, education, and/or nursing. Originally conceived as one person filling the role of champion, one FQHC organization has implemented a Champion Distributive Model whereby one individual is identified as the lead champion with other team members contributing supporting expertise such as quality improvement, outreach, or education.

Thus, the cancer control champion is pivotal to the CARES-REACH intervention at each of the three levels: 1) organization or systems level to prioritize CRC screening and optimize EMR systems for implementation of CRC screening; 2) provider education (initial and annual booster training) and feedback to support evidence-based CRC screening patient education, recommendation and distribution of FIT kits; and 3) patient level (coaching and addressing patient-specific barriers for individuals who do not return a FIT kit within 90 days of distribution). This latter level represents an approach supported by increasing evidence that is predicated on patient activation, education, and support for promoting both initial and repeat screening (15–26, 36, 54–57). Our evaluation efforts include understanding the infrastructure, resources, and processes for role sustainability.

For patients who receive a FIT kit or recommendation for other screening modality and have not completed any screening at 3-month EMR review, a coach intervention will be implemented by the cancer control champion. Coaching has been shown to increase engagement and improve outcomes (58). This component, delivered primarily by telephone (estimated 10 minutes), is a personalized modality intended to 1) assess the patient’s test-specific barriers; 2) assist patients in understanding the importance of repeat screening through education and use of materials; 3) coach the patient on personalized strategies to overcome their stated barriers; and 4) promote a sense of empowerment to use the information and complete a FIT screening. For individuals who continue to have questions or unresolved barriers, the coach will refer them to their provider for further management.

A multilevel approach to deliver EBIs using a stepped wedge design with one year of observation is being employed to observe secular trends followed by a sequential roll-out of the intervention using two waves, allowing for an extension of at least two years after intervention initiation, which allows for assessing screening maintenance. The stepped wedge approach aims to accelerate the rollout of EBIs in “real world” practice settings while capturing the added effect of a Cancer Control Champion. The multilevel approach expedites the successful transfer of an intervention to other settings as the design is intended to disentangle contextual factors that may affect optimal implementation. The main outcome assessed is a change in annual screening rates (all screening modalities), and secondary measures include sustained maintenance.

CFIR is a determinant framework focused on settings, barriers, and facilitators. Therefore, CFIR constructs that inform the current study include external CRC screening benchmark requirements, organizational readiness, compatibility, patient characteristics, adopter [provider, clinic] characteristics, engagement, cost, and cancer control champion involvement (44).

### Measures

Through process evaluation, the CARES-REACH program implementation will be assessed to ensure it is proceeding as planned (e.g., meeting the goals and timelines for accomplishing each component of the program implementation). Guided primarily by the CFIR and RE-AIM frameworks, we developed and implemented a Readiness Evaluation Assessment Level (REAL) survey with FQHC institutional leaders, cancer control champions, and a diverse group of providers (e.g., medical assistants, nurses, Advance Practice Practitioners [ARNP/PA], and physicians) from various clinic locations to solicit feedback about how to assess implementation processes including facilitators/impediments of intervention implementation, at three time points (baseline, mid-point and at the end of project implementation). This questionnaire solicits readiness feedback from leaders, providers, and champions (key stakeholders involved in the implementation of CRC screenings) from our two partnering FQHC organizations at three timepoints. The information gained will provide context for study findings (i.e., screening uptake and trends) and contribute to sustainability planning, scale-up, and maintenance. Through this process and conclusions, we will continually refine and improve processes and determine whether the current approaches effectively bring about the desired outcome (i.e., improvements in clinic CRC screening rates).

Notably, the significance of the proposed work for FQHC systems is potentially high for improving their overall UDS screening measures (annual rates) over time. UDS is a crucial quality and performance measure in primary care used by FQHCs to calculate the percentage of patients receiving a recommended (prevention) care service, such as CRC screening [94]. According to recent data from our two participating FQHC organizations (Central Florida Health Care (CFHC) and MCR Health (MCR)), organization-wide UDS measures from 2015 to 2017 ranged from 26–38%, indicating that a unique window of opportunity exists for improvement. Table 5 outlines the constructs and outcome metrics aligned with the RE-AIM and CFIR implementation science frameworks.

### Statistical and Data Analysis

A longitudinal linear mixed model with 3-year CRC screening rates will be used to analyze data for Aim 1. The linear mixed model to assess changes in UDS will include 42 (= 14 clinics x 3 years) CRC screening rates due to the stepped wedge randomization of these clinics. The model will use independent variables: intervention (fixed), clinic (random), rural (urban = reference group; fixed), and time (fixed), using an unstructured variance-covariance model. The model is expected to provide an estimate of the secular time trend of about 2.7% per year on a national level (in 2014–2016). For the stepped-wedge portion of the study, we will have 83.6% and 97.5% power to detect a difference of 4% and 5% in the CRC screening rates between intervention and non-intervention clinics, assuming an intracluster correlation coefficient (ICC) of 0.013, where the power is minimized (obtained from PASS15).

For Aim 2, the analysis will include a logistic regression model, with successful screening as the dependent variable. The independent variables will consist of patient, practice, and organization. Data for all patients seen at participating clinics in a given calendar year will be analyzed by year and clinic setting to examine trends in screening rates at the individual patient level. To show their screening rate patterns, we will ascertain individual-level data with a non-PHI patient ID. We will also estimate the proportion of FIT-screened patients who complete FIT screening in the intervention’s initial wave (intervention vs. control) and subsequent year(s). We will estimate the probability of initiating screening in years 2 and 3 for patients seen in the clinic but not screened in previous years.

For aim 3, we will evaluate the intervention’s success and how to expand its reach to other community health systems. The RE-AIM (59) and CFIR (60) frameworks will serve as guides for this evaluation of the intervention’s implementation process (organization-wide implementation experience) and resource requirements. The implementation process will be analyzed using both qualitative and quantitative methods. Through process evaluation, we will assess whether the CARES-REACH study implementation is proceeding as planned through tracking documentation, detailing process changes, and regular champion interactions.

## RESULTS

Study initiation has begun and is in progress. As such, implementation and data collection is ongoing. However, at the study’s conclusion, and when successfully implemented, the following data are anticipated: overall organization and clinic-specific UDS rates, FIT and other CRC screening completion rates, and patient reminders and coaching efforts. Additionally, cost and resource use, characteristics of the FQHCs and their staff, champion involvement and sustainability, and process modifications will be tracked and analyzed.

## DISCUSSION

Implementation of CARES-REACH in FQHCs sets the stage for a CRC screening community intervention with high potential for sustainability in rural and urban settings to meet and accelerate the achievement of current guidelines and goals. By extending this model at the regional, state, and national levels through FQHC networks and similar practice settings, CARES-REACH contributes to cumulative evidence and advancement of cancer control practice guidelines. Additionally, the cancer control champion model could be extended beyond CRC screening to additional cancer prevention, chronic disease management, and care delivery to impact health disparities and advance health equity. The study is in progress, and implementation of both waves has been initiated. Our Cancer Control Champions are in place in their respective FQHC organizations. Collectively, the champion(s) are currently working to promote CRC screenings, interact with providers, monitor organizational EMR processes, and coach and navigate patients. Providers and staff have attended education and training sessions to assist in identifying patients more effectively who need CRC screening, improve ways for ordering and recommending screening tests, and enhance patient monitoring after receiving a screening test. We anticipate that successful completion of the CARES-REACH study will inform organizations, providers, researchers, and policymakers on strategies to adapt, facilitate, and guide organizational implementation of cost-effective EBIs that can significantly transform the organization’s cancer screening measures, bringing sustained benefit to patients and contribute to overall improved community health.

## Figures and Tables

**Figure 1:**
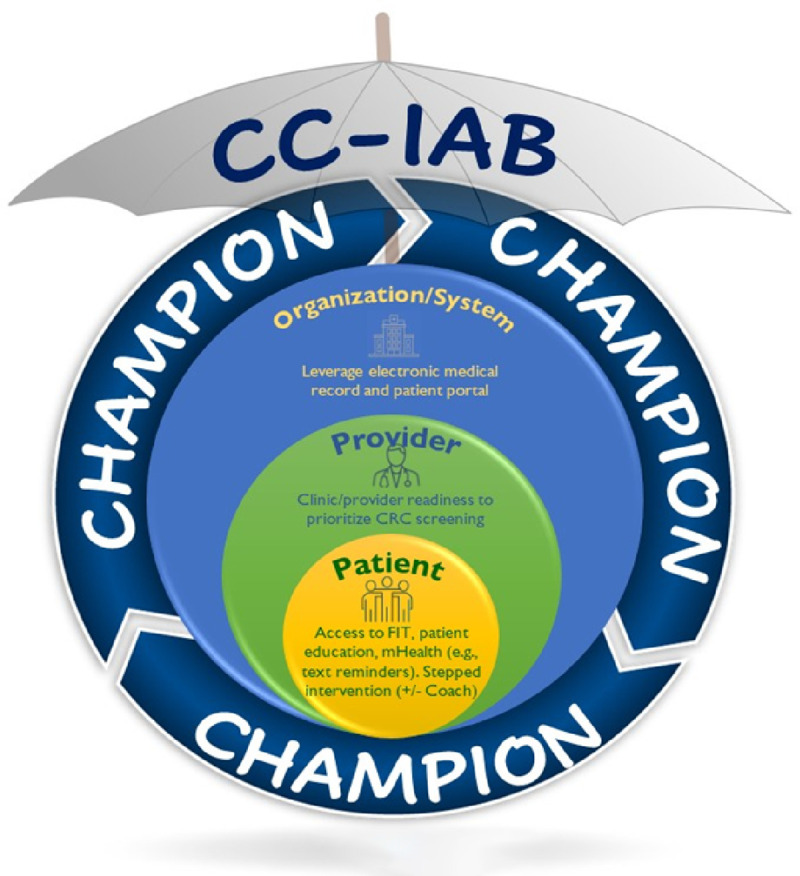
Legend not included with this version.

**Table 1:** Intervention Strategy Examples

Level	Intervention Strategy Examples
**Organization**	Optimizing EMR Processes (Automated Queries, Flags, provider/nursing prompts and reminders, etc.)
	Automated Calls, Texts, Patient Portals- messages sent to Patients about CRCS (Recipient = Patients)
	Wave 1 Clinics Activated - intervention implementation at all three levels.
	Wave 2 Clinics Activated - intervention implementation at all three levels.
	Readiness Evaluation Assessment Level (REAL) Survey Measure – at baseline and repeated at the midpoint and end of the implementation
	CRC Awareness Activities (i.e., CRC awareness month, promotional items, etc., throughout the project period)
**Provider**	Initial Provider Education (physicians, nurses, staff) – by wave of stepped-wedge and rollout design
	Annual Booster Education – by wave of stepped-wedge and rollout design
	Providers engaging with Champion (e.g., CRC screening scorecards)
	REAL Survey Measure (baseline and repeated at midpoint and end of implementation)
**Patients**	Dissemination of Language-Specific Educational Materials (Recipient: Patients)
	Patients Receive FIT kits (and other appropriate screening modalities) Coaching of patients who do not complete prescribed screening: From Champion (at 30 days or as needed; (Recipient = Patients)
**Champion**	Champion Hire/trained. 1 Champion for each FQHC/Organization level
	Assist in Educating and Training Providers (Physicians, Nurses, Medical Assistants, staff) (Target: Providers)
	Assist in Optimizing EMR (Target: Organization)
	Identify Patients who don’t return FIT (Recipient: Patients)
	Coach Intervention and Navigate Patients (Recipient: Patients)
	Organizational Change Agent (Recipient: Organization, Provider, and Patient Levels) Assist in identifying patients needing screening (EMR/Organization and provider levels) and identify individual patients needing coaching (patient level)
	REAL Survey Measure (repeated from baseline, midpoint, and end of project implementation

**Table 2:** Stepped Wedge Design with Extension for Maintenance:

	Year 0 Baseline	Year 1 Wave 1	Year 2 Wave 2	Year 3 Sustaining	Year 4 Evaluation
Features/Aim	(Pre-Intervention)	Initiation	Initiation	Implementation	
**Number of Clinics**	0/14	7/7	14/0	14/0	14/0
**Exploration of EMR Optimization**	Yes	Yes	Yes	Yes	Yes
**Provider Education and EBIs for Patients**	N/A	Yes	Yes	Yes	Yes
**Coach Intervention**	N/A	Yes	Yes	Yes	Yes
**Annual Provider Booster Education**	N/A	N/A	Yes	Yes	Yes

**Table 3:** CARES-REACH Aims

	Aim	Expected Outcomes
**Aim 1**:	Implement an organization-wide multilevel CRC screening intervention using evidence-based interventions (EBIs) and an organization-wide cancer control champion in FQHCs in rural and urban Central and Southwest Florida.	The primary outcome measured is the change in annual CRC screening rates (UDS) at the organization and intervention clinic sites, among other screening uptake behaviors.
**Aim 2**:	Explore whether practice setting (rural vs. urban) and population characteristics (e.g., nativity [foreign-born status], language preference, education, income, health insurance, etc.) may differentially impact annual clinic CRC UDS rates and other screening outcomes.	Patient de-identified data will be pooled for analysis. This method will be useful in determining whether or not differences can be attributed to factors outside of the typical clinical setting. This aim is to explore disparities by geography and population characteristics.
**Aim 3**:	Conduct a comprehensive evaluation of the implementation process, resource requirements, and intermediate patient screening outcomes of the CARES-REACH program in FQHCs.	To achieve this objective, we will undertake a comprehensive evaluation of the CARES-REACH program (facilitators/obstacles) to inform and guide program adjustments and upgrades at the two existing FQHC systems. If these assessments successfully yield informative data, more community and FQHC locations may be able to implement the CARES-REACH program thanks to this method (scale-up).

**Table 4:** Multi-Level Activities

Champions		

Complete REAL survey (baseline, midpoint/ first follow up, second follow up). Assist in the Education and training of providers. Assist in the optimization of EMR for CRC screening. Identify patients who do not return the FIT Kit Coach and navigate patients. Provide feedback to patients and providers. Serve as Organizational change agent for cancer screening. Facilitate activities at Organization/Systems, Provider, and Patient levels.		

Organization/Systems	Provider	Patient

Complete REAL survey (baseline, midpoint/ first follow-up, second follow-up) Optimize EMR processes. Examines screening status queries at the organization, clinic site, and provider levels. Provides provider-tailored feedback and support to improve rates.	Complete REAL survey (baseline, midpoint/ first follow up, second follow up). Complete CRC training. Prioritize CRC screening. Continue usual CRC screening and recommendation. Assist in CRC screening and CRC screening education, and feedback with provider scorecards (screening rates per provider). Provide linkage to care.	Receive education. Return FIT kits and complete other CRC screening modalities as indicated. Receive tailored coaching as needed. Complete colonoscopy referrals as needed.

**Table 5:** Assessment of Implementation Science Constructs

Implementation Framework/Constructs	Examples of data elements	Time points
**RE-AIM**		
**Reach**	- Number (and %) of patients who receive intervention materials. - Number (and %) of providers/staff who receive initial education. - Number (and %) of providers/staff who receive booster education. - Number (and %) of patients identified as being applicable for coaching and who receive coaching. - Number of patient reminders sent.	Years 1–5
**Effectiveness**	- Overall clinic UDS rates. - Number (and %) of patients who complete initial FIT. - Number (and %) of patients who complete repeat FIT.	Years 1–5
**Adoption**	- Provider use of IT query feature. - Provider CRC screening order rates. - Use of communication methods (e.g., text, patient portal, automated calls) to send patient reminders.	Years 1–5
**Implementation**	- Completion of EMR enhancements. - Number of education sessions completed.	Years 1–5
**Maintenance/ Sustainability**	- Number (and %) of patients who repeat FIT.	Years 2–5
**CFIR**		
**Intervention characteristics**	- Cost, resource use assessment.	Years 1–5
**Inner setting**	- FQHC characteristics. - Organizational readiness. - Compatibility with organizational priorities.	Years 1–5
**Outer setting**	- External CRC screening benchmark requirements (UDS); National Targets per CDC and National Colorectal Cancer Roundtable; Florida CRC screening rates.	Years 1–5
**Characteristics of individuals involved**	- Adopter [provider, clinic] Characteristics.	Ongoing
**Process**	- Champion involvement and process modifications.	Years 1–5

## Data Availability

Not applicable (this manuscript does not report data generation or analysis)
